# The structure of microbial populations in Nelore GIT reveals inter-dependency of methanogens in feces and rumen

**DOI:** 10.1186/s40104-019-0422-x

**Published:** 2020-02-24

**Authors:** Bruno G. N. Andrade, Flavia A. Bressani, Rafael R. C. Cuadrat, Polyana C. Tizioto, Priscila S. N. de Oliveira, Gerson B. Mourão, Luiz L. Coutinho, James M. Reecy, James E. Koltes, Paul Walsh, Alexandre Berndt, Julio C. P. Palhares, Luciana C. A. Regitano

**Affiliations:** 1 Embrapa Southeast Livestock, São Carlos, Brazil; 2 Department of Molecular Epidemiology, German Institute of Human Nutrition Potsdam-Rehbrücke (DIfE), Nuthetal, Germany; 3 NGS Genomic Solutions, Piracicaba, Brazil; 4 Department of Animal Science, University of São Paulo/ESALQ, Piracicaba, Brazil; 5 Department of Animal Science, Iowa State University, Ames, IA USA; 6 NSilico Life Science, Dublin, Ireland

**Keywords:** Archaea, Bacteria, *Bos indicus*, Metabarcoding, *Methanobrevibacter*, Microbiome, Microbiota

## Abstract

**Background:**

The success of different species of ruminants in the colonization of a diverse range of environments is due to their ability to digest and absorb nutrients from cellulose, a complex polysaccharide found in leaves and grass. Ruminants rely on a complex and diverse microbial community, or microbiota, in a unique compartment known as the rumen to break down this polysaccharide. Changes in microbial populations of the rumen can affect the host’s development, health, and productivity. However, accessing the rumen is stressful for the animal. Therefore, the development and use of alternative sampling methods are needed if this technique is to be routinely used in cattle breeding. To this end, we tested if the fecal microbiome could be used as a proxy for the rumen microbiome due to its accessibility. We investigated the taxonomic composition, diversity and inter-relations of two different GIT compartments, rumen and feces, of 26 Nelore (*Bos indicus*) bulls, using Next Generation Sequencing (NGS) metabarcoding of bacteria, archaea and ciliate protozoa.

**Results:**

We identified 4265 Amplicon Sequence Variants (ASVs) from bacteria, 571 from archaea, and 107 from protozoa, of which 143 (96 bacteria and 47 archaea) were found common between both microbiomes. The most prominent bacterial phyla identified were Bacteroidetes (41.48%) and Firmicutes (56.86%) in the ruminal and fecal microbiomes, respectively, with *Prevotella* and Ruminococcaceae UCG-005 the most relatively abundant genera identified in each microbiome. The most abundant archaeal phylum identified was Euryarchaeota, of which *Methanobrevibacter gottschalkii*, a methanogen, was the prevalent archaeal species identified in both microbiomes. Protozoa were found exclusively identified in the rumen with *Bozasella*/*Triplumaria* being the most frequent genus identified. Co-occurrence among ruminal and fecal ASVs reinforces the relationship of microorganisms within a biological niche. Furthermore, the co-occurrence of shared archaeal ASVs between microbiomes indicates a dependency of the predominant fecal methanogen population on the rumen population.

**Conclusions:**

Co-occurring microorganisms were identified within the rumen and fecal microbiomes, which revealed a strong association and inter-dependency between bacterial, archaeal and protozoan populations of the same microbiome. The archaeal ASVs identified as co-occurring between GIT compartments corresponded to the methanogenic genera *Methanobrevibacter* and *Methanosphaera* and represented 26.34% of the overall archaeal sequencesdiversity in the rumen and 42.73% in feces. Considering that these archaeal ASVs corresponded to a significant part of the overall diversity of both microbiomes, which is much higher if one includes the interactions of these co-occurring with other rumen archaea ASVs, we suggest that fecal methanogens could be used as a proxy of ruminal methanogens.

## Background

Nelore (*Bos indicus*) is a beef breed adapted to tropical environments and constitutes most of the biggest commercial herd in the world, the Brazilian bovine herd. Beef production is a fundamental part of the Brazilian economy. It represents 6% of the gross domestic product (GDP), with more than 1.6 million tons of beef exported worldwide in 2018 (http://www.abiec.com.br).

Ruminants, such as Nelore, require an abundant and diverse ruminal microbiota in order to digest complex polysaccharides, such as cellulose. This community produces enzymes capable of breaking down these polysaccharides into short-chain fatty acids (SCFAs), that provide the host with nutrients and energy required for its development and maintenance through enteric fermentation [1, 2]. Fermentation also produces methane as a by-product, which is a greenhouse gas that contributes 28 times more to climate change than carbon dioxide, through the action of methanogenic archaea [3], also known as methanogens. These microorganisms are responsible for the production of 7–18% of greenhouse gases of anthropogenic origin and the loss of up to 2–12% of the total energy ingested, therefore, also negatively affecting animal productivity [4–6].

Several studies that aimed to characterize the gastrointestinal tract (GIT) microbiota and its genetic material, the microbiome, of ruminants using culture-independent approaches have been published over the years, including studies of Brazilian Nelore cattle [7–9]. One limitation to these studies is the difficulty in sampling rumen digesta in the routine of cattle breeding, which can be achieved only by oral intubation, rumenocentesis, fistulation, or after slaughter. These methods are not conducive to effectively monitoring the cattle microbiome without generating stress or compromising the production system. Alternatives, such as the use of oral samples as a proxy for the rumen microbiome, have been proposed [10] and shown to be good predictors for the bacteria community, but not for archaea. Other sampling alternatives for monitoring the GIT microbiome, such as colon/fecal are poorly investigated or limited to a small number of animals [11], despite being identified as crucial in other mammals [12–14].

To fill some of these gaps and to propose new sampling strategies, this study aimed to characterize microbial populations from two distant sections of the Nelore GIT, the rumen and rectal ampulla, and to search for co-occurring patterns within and between these microbiomes.

## Methods

### Experimental design, sample collection and processing

Fecal and ruminal samples were collected from a population of 26 Brazilian Nelore bulls, born in 2014 and slaughtered in 2016, with an average age of 1 year and 11 months. The experiment was carried out at Embrapa Southeast Livestock and lasted 105 days, of which 15 days were exclusively utilized for animal adaptation to the feedlot. The diet consisted of corn silage (82%), corn grains (11.83%), soy grains (4.69%), mineral supplements (1.48%), active dry yeast, virginiamycin and monensin. Approximately 10 g of feces was collected from the rectal ampulla of each animal two weeks before slaughtering, kept on ice for approximately 2 h and stored at − 80 °C. About 50 mL of rumen content was collected from each animal immediately after slaughter, frozen in liquid nitrogen and stored at − 80 °C.

DNA extractions from ruminal and fecal samples were performed by using the Quick-DNA™ Fecal/Soil Microbe Miniprep Kit (ZYMO Research Corp., Irvine, CA) using 150 mg of each sample, as stated by the standard protocol. PCR amplification of the bacterial and archaeal 16S rRNA genes and protozoa 18S rRNA gene were performed with the primers set described in Table 1. Additionally, barcode indexes were added to all samples to allow for sample multiplexing. Amplicons were pooled at equimolar ratios in sequencing libraries and sequenced by an Illumina MiSeq platform (2 × 250 bp) using the Illumina V3 sequencing kit at the ESALQ Genomics Center (Piracicaba, SP, Brazil). The sequence data is available from the Sequence read archive (SRA) [accession number PRJNA525838].

**Table 1:** Primer set used to amplify 16S and 18S hypervariable regions of Bacteria, Archaea and ciliate Protozoa

Identifier	Sequence	Target	Reference
341-b-S-17F	CCTACGGGNGGCWGCAG	Bacteria	[15]
785-a-A-21R	GACTACHVGGGTATCTAATCC	Bacteria	[15]
Ar915aF	AGGAATTGGCGGGGGAGCAC	Archaea	[16]
Ar1386R	GCGGTGTGTGCAAGGAGC	Archaea	[16]
Reg1320R	AATTGCAAAGATCTATCCC	Protozoa	[2]
RP841F	GACTAGGGATTGGARTGG	Protozoa	[2]

### Data pre-processing and analysis

Raw reads were quality checked, filtered for quality (>Q25) and trimmed at the positions 220 (forward) and 175 (reverse) based on aggregated quality plots generated by QIIME 2 (version 2018.8) [17]. The remaining data were submitted to DADA2 to resolve the sequences into Amplicon Sequence Variants (ASVs), instead of traditional Operational taxonomic units (OTUs) [18], which presents an improved taxonomic resolution and consistency. Additionally, chimeric sequences were excluded using the DADA2 algorithm.

Bacterial sequences were classified using the SILVA database version 132 [19], archaeal sequences using the Rumen and Intestinal Methanogen database (RIM-DB) [20] and protozoa sequences using a curated database [21]. Rarefaction curves were generated for each dataset and used to standardize the data (Additional file 1: Figure S1A, Additional file 2: Figure S2A). The resulting ASV table was used to determine alpha (Number of ASVs and Shannon-Wiener index) and beta diversities (Unweighted Unifrac distance) with QIIME 2.

### Statistical analysis

In order to identify differences in the microbial community structure, alpha and beta diversities were contrasted using nonparametric statistical methods Kruskal-Wallis and PERMANOVA, respectively.

To be able to identify co-occurrence patterns among microorganisms, the correlation between their abundances was inferred using the SparCC algorithm, a statistical approach developed for sparse and compositional data, with 1000 bootstrap replicates [22, 23]. This algorithm applies a Bayesian model to estimate the actual fractions from observed counts and infer the Pearson correlation of log-ratio variances of ASV fractions. To avoid spurious correlations, only ASVs identified in 10% of the samples with at least 100 sequences in total and |r| > 0.6 were considered for intra microbiome analysis. Also, due to the reduced number of ASVs common to both microbiomes, only those with 10 sequences in total and |r| > 0.5 were considered for the inter microbiome analysis. Principal coordinate analysis (PCoA) with unweighted unifrac distance was performed by QIIME 2 and used to identify variables that stratify the samples. Co-occurrence networks were generated using the software Cytoscape [24].

## Results

### Microbiome composition

Sequencing of bacterial, archaeal, and protozoa amplicons from the rumen and fecal samples of 26 animals yielded a total of 9,667,533 paired-end reads (5,361,879 paired-end reads for bacteria, 2,706,672 for archaea and 1,598,982 for protozoa). Quality control, denoising and chimera exclusion retained a total of 5,465,431 sequences resolved in 13,680 ASVs. A total of 4943 ASVs (4265 for the bacteria, 571 for the archaea and 107 for protozoa datasets) were retained after the exclusion of singletons. Among the bacterial and archaeal ASVs identified, 143 were common to both microbiomes, which comprised 96/4265 (2.30%) for the bacteria and 47/571 (8.23%) for the archaea datasets. Rarefaction curves based on the alpha-diversity metrics of Shannon-Wiener (diversity) reached a plateau, which indicated that the sampling depth was adequate, and additional sequences would not likely result in additional features.

We were not able to identify protozoa 18S rRNA amplicons in the fecal microbiome; therefore, the following analyses were performed exclusively on the bacteria and archaea datasets. To be able to compare the diversity of the microbiomes, the data was rarefied to 40,000 reads for the bacteria dataset and to 6000 reads for the archaea dataset. Comparisons between microbiomes using alpha-diversity metrics (Number of ASVs and Shannon-Wiener indexes) revealed the bacterial diversity of the rumen microbiome to be significantly richer and diverse than the fecal microbiome (*P* < 0.01) (Additional file 1: Figure S1B, C, Additional file 2: Figure S2B, C). We found no significant difference between the number of archaeal ASVs in the rumen and fecal microbiomes. However, we found a significant difference between the Shannon-Wiener index of both environments, with the ruminal archaea population richer than the fecal population.

Moreover, comparisons of beta-diversity metric (Unweighted Unifrac distance), performed with Principal coordinates analysis (PCoA) and PERMANOVA, showed two spatially separated and significant clusters (adjusted *P* < 0.01) that corresponded to the rumen and fecal microbiomes (Additional file 1: Figure S1D, Additional file 2: Figure S2D).

### Taxonomic composition of bacteria in ruminal and fecal microbiomes

A total of 19 phylum, 31 classes, 55 orders, 93 families and 222 genera were identified in the rumen and fecal microbiomes. At the phylum level, Bacteroidetes (41.94% ± 3.39%) was the most relatively abundant population in the rumen microbiome, followed by Firmicutes (36.81% ± 2.90%), Proteobacteria (6.28% ± 3.51%) and Spirochaetes (4.80% ± 1.41%). Conversely, Firmicutes was the most abundant phylum in fecal microbiomes (56.67% ± 5.08%), followed by Bacteroidetes (27.65% ± 3.96%), Proteobacteria (11.76% ± 6.21%) and Tenericutes (1.50% ± 1.02%).

**Fig. 1:**
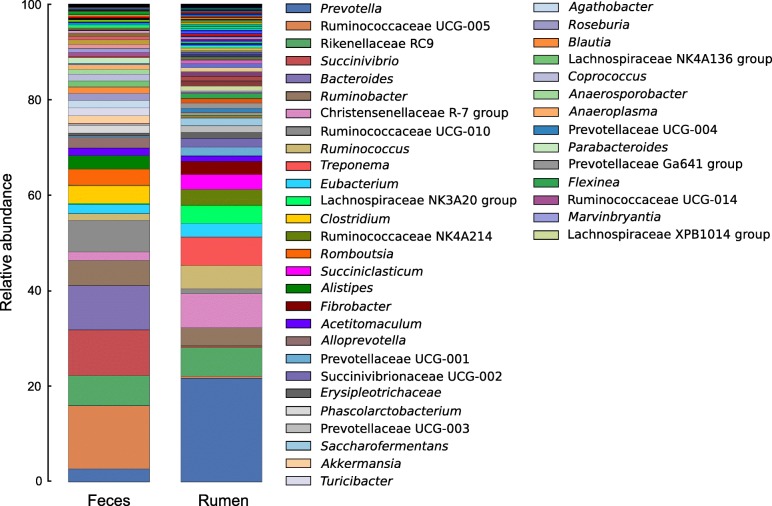
Relative abundance of bacterial populations identified in rumen and feces of Nelore. The percentage is shown on the Y-axis. Only microorganisms with a relative abundance higher than 0.5% are represented in the legend

At the genus level, *Prevotella* (16.81% ± 3.54%), Christensenellaceae R-7 (5.59% ± 1.55%), Rikenellaceae RC9 (4.8 ± 1.50%) and *Treponema* (4.40% ± 1.50%) were the most relatively abundant in the rumen microbiome. Conversely, the genera Ruminococcaceae UCG-005 (10.85% ± 3.68%), *Succinivibrio* (7.86% ± 2.71%), *Bacteroides* (7.76% ± 1.79%), Ruminococcaceae UCG-010 (5.52% ± 2.42%) and Rikenellaceae RC9 (5.11% ± 1.11%) were the most abundant in the fecal microbiome (Fig. 1).

### Taxonomic composition of archaea in ruminal and fecal microbiomes

The archaeal diversity of both microbiomes was limited when compared to the bacterial community. It harbored a total of one phylum, three classes, three orders, three families, four genera and seven species. At the phylum level, Euryarchaeota was the only phylum identified in all samples using RIM-DB.

At the genus level, the rumen microbiome was populated by *Methanobrevibacter* (69.46% ± 3.73%), *Methanosphaera* (2.91% ± 0.76%), *Methanomicrobium* (0.65% ± 1.10%), and an uncultured genus from the Methanomassiliicoccaceae family (22.85% ± 3.65%). The fecal microbiome was populated by *Methanobrevibacter* (88.36% ± 2.56%) and *Methanosphaera* (1.39% ± 0.67%), being less diverse than the rumen microbiome. RIM-DB allowed us to assign ASVs identified as *Methanobrevibacter* into two dominant species clades, the *Methanobrevibacter gottschalkii* and *Methanobrevibacter ruminantium*, as well as other species of this genus, such as *Methanobrevibacter smithii* and *Methanobrevibacter wolinii*. These represent most of the archaeal diversity in the rumen (49.67% for *M. gottschalkii*, 18.37% for *M. ruminantium*) and fecal microbiomes (76.96% for *M. gottschalkii*, 9.70% for *M. ruminantium*).

**Fig. 2:**
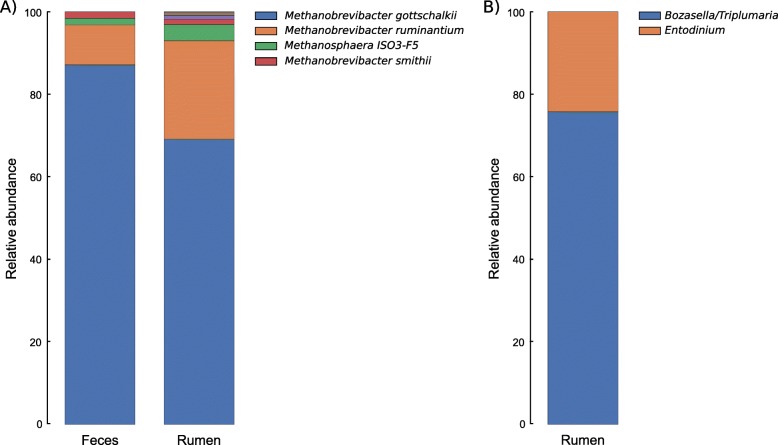
**a** Relative abundance of archaea populations identified in rumen and feces of Nelore. **b** The relative abundance of protozoan populations identified in the rumen of Nelore. The percentage is shown on the Y-axis. Only microorganisms with a relative abundance greater than 0.5% are represented in the legend

Additionally, 47 out of 571 archaeal ASVs identified as common to both microbiomes represented part of the of the *M. gottschalkii, M. ruminantium* and *Methanosphaera* populations. These ASVs corresponded to 54.63%, 36.62% and 82.58% of these microorganisms’ diversities in the rumen and 64.03%, 39.91% and 84.72% in the fecal microbiomes (Fig. 2a).

### Taxonomic composition of ciliate Protozoa

The protozoa community of Nelore harbored a single phylum and class, two orders, three families and five genera. We identified the genus *Bozasella/Triplumaria* as the most abundant (45.67% ± 30.17%), with a relative abundance that ranged from 5.53% to 84.47% in the 26 rumen samples. The genus *Entodinium* was the second most abundant and prevalent genera, followed by uncharacterized protozoa from the Ophryoscolecidae and Isotrichidae families (Fig. 2b). The other families and genera accounted for less than 0.05% of the mean relative abundance.

### Co-occurrence patterns among microbial populations

Correlations between the abundance patterns within and between bacteria, archaea and ciliate protozoa communities were tested to evaluate if these populations were interdependent, considering both the |r| threshold and significance defined in the methods section. A total of 1703 and 952 co-occurrences were identified in the fecal and ruminal microbiomes, respectively. These co-occurrences were used to generate co-occurrence networks (Additional file 2: Figure S2, Additional file 3: Figure S3, Additional file 4: Figure S4).

**Fig. 3:**
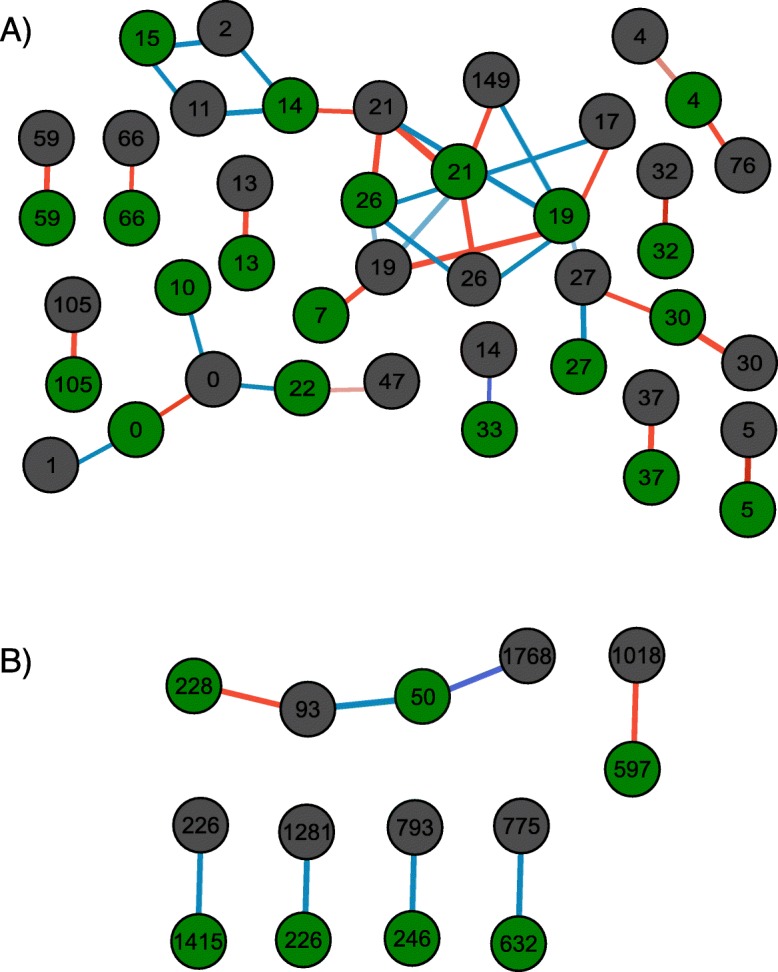
Co-occurrence networks of ASVs common to ruminal (Green nodes) and fecal (Gray nodes) microbiomes, based on SparCC results. ASVs are represented by their respective numbers, with red edges representing positive correlation and blue, negative. **a** Eleven co-abundance networks with archaeal ASVs showing 12 positive correlations between the same ASV in different microbiomes. **b** Co-abundance networks with bacterial ASVs showing two positive correlations between the fecal and ruminal bacteria

Besides, 39 significant co-occurrences between ruminal and fecal archaea, of which nine identified as *M. gottschalkii*, one as *M. ruminantium* and three as *Methanosphaera*, co-occurred with the same ASV in the fecal microbiome (Fig. 3a). Conversely, eight significant co-occurrences between ruminal and fecal bacteria (Fig. 3b) were identified, but none between the same bacterial ASV.

## Discussion

In recent years, culture-independent approaches have been applied to investigate microbial populations from the GIT of ruminants. Herein, we investigated the microbiome located in different sections of the Nelore cattle GIT, including bacteria, archaea and protozoa, and the relationship between these microorganisms, using metabarcoding. This study also characterized the distribution, relatedness, and co-occurrence of microbial populations within and between fecal and ruminal microbiomes.

### The structure of Nelore rumen and fecal microbiomes

Our results revealed that the ruminal and fecal microbiomes in Nelore cattle are significantly different based on both alpha and beta diversities. These results are in agreement with previous studies that reported the microbiome composition of GIT segments of bovines [10, 14, 25]. Analysis of alpha-diversity metrics also revealed that the Nelore cattle rumen microbiome was richer and more diverse when compared to the fecal microbiome, which confirms previous results with Nelore cattle [9].

The Phylum Bacteroidetes dominated the Nelore rumen community (41.65%), consistent with results from *Bos taurus* breeds such as Angus and Holstein, and other ruminant species [5, 25, 26]. Firmicutes (36.81%) and Proteobacteria (6.27%) also comprised a large proportion of the rumen microbiome. Differences in the ratio between Bacteroidetes and Firmicutes have been associated with obesity in humans [27] and milk-fat yield in Holstein Friesian cows [5]. At the genus level, *Prevotella*, a genus commonly found in the GIT of mammals and a significant player in carbohydrate metabolism and cellulose/hemicellulose degradation in ruminants, was identified as the most abundant genus, followed by Rikenellaceae RC9 and Christensenellaceae R-7, both previously reported in ruminants [28].

Firmicutes, the most prevalent phylum in the fecal microbiome, was represented primarily by the genera Ruminococcaceae UCG-005 and UCG-010, as previously described for post-weaning Holstein calves, Ayrshire cows and wild forest musk deer fecal microbiomes [10, 29, 30]. A single and abundant ASV, identified as *Succinivibrio*, was found in a substantially higher abundance in the fecal (7.86%) than in the ruminal microbiome (0.23%) of Nelore. This genus, of which the only described species is the *Succinivibrio dextrinosolvens*, is comprised of a rod-shaped bacterium that contributes to rumen starch digestion through the degradation of glucose. Furthermore, this genus is abundant in animals that are fed high starch diets [31].

The genus *Methanobrevibacter* was the most abundant archaea identified in both microbiomes, with the clades *M. gottschalkii* and *M. ruminantium* as the most prevalent. Strictly anaerobic microorganisms belonging to the archaea domain represent a small proportion of the overall taxonomic diversity in the rumen microbiome [26, 32, 33]. Most species of methanogenic archaea can use hydrogen, formate and methyl-compounds as their primary energy source, thereby reducing CO_2_ to CH_4_ in the rumen fermentation process.

Previous studies have shown that, rather than the absolute number of archaea, the contribution of individual methanogenic species is essential for CH_4_ production, especially the abundance of *M. gottschalkii* and *M. ruminantium* [34]. Recently, a study compared two groups of cows that were divergent for methane emission and found a higher relative abundance of *M. gottschalkii* in cows that were high CH_4_ emitters and of *M. ruminantium* in cows that were low emitters [34]. The authors suggested that this association could be due to differences in fermentation patterns of these methanogenic clades. Indeed, *M. ruminantium* M1 genome lacks the coding genes for methyl-CoM reductase II (McrII), having only genes for the isomeric form McrI [35]. McrI is expressed in low levels of H_2_ [36], which indicates that *M. ruminantium* is suited to thrive in an environment with low concentrations of H_2_, while *M. gottschalkii* is suited for high concentrations of H_2_.

Additionally, the genus *Methanocorpusculum* was not detected in any of the fecal samples used in this work, despite being identified (52%) in the feces of five Ayrshire cows (52%) [10] and Holstein cows (10.38 to 39.24%) [36]. The addition of supplements, such as dry yeast, seems to play a negative role in the abundance of this genus in dairy cattle [37], which could contribute to its absence in our Nelore cattle microbiome. Thus, considering the scarce data on bovine fecal archaeal microbiome, our results reinforce the high dependency of the microbiome profile on breed, feed and environmental factors.

Regarding the rumen protozoa microbiome, the most abundant and prevalent genera identified in Nelore was the combination of the genera *Bozasella* and *Triplumaria*, because their respective 18S rRNA are 100% identical. Both genera have been identified in Asian and African elephant intestines [38, 39]. Recently, these genera were reclassified to the unclassified family 1 (Uncf1) by Henderson et al and were not identified in the biggest survey of rumen microbiomes so far [2]. The reduced number of representative sequences in databases at the time the study was performed can explain this lack of results, or even the use of additives, such as monensin [40]. Also, the genus *Entodinium*, identified as the dominant protozoan genus in previous work and widespread in ruminants, was identified as the second most abundant genera and was present in all rumen samples analyzed in this study.

Although not essential for host survival, the rumen ciliate protozoa community is responsible for the release of vast amounts of hydrogen, contributing to methanogenic archaea metabolism and, therefore, methane production [41]. In fact, methane production drops by 11% when the host is defaunated, a process that removes the protozoa community, which can be taken as evidence of their importance in the methane production process [42].

### Co-occurrence of microbial populations suggests a close relationship among fibrolytic bacteria and methanogenic archaea

Among the co-occurrent microorganisms, most are expected to be linked by metabolic processes, either synergistically in a mutualistic relationship, or through competition with organisms that share the same biological niche. For example, the ruminal ASV 33, classified as *Succiniclasticum*, a genus comprised by a single bacterium that ferments succinate into propionate, presented 11 connections in the co-occurrence network (3 positives, eight negatives). This ASV is co-occurring with ASVs identified as *Prevotella*, the most abundant genus in the rumen environment and a producer of succinate through the degradation of polysaccharides and glucose [43, 44]. In contrast, ASV 33 had a negative co-occurrence with three ASVs identified as *Muribaculaceae* (SparCC < − 0.6), a family of uncharacterized genera that are highly associated with the increase of propionate concentrations from succinate [45]. Thus, their negative correlation could be due to competition for substrates and nutrients.

The fecal ASV 1, classified as Ruminococcaceae UCG-005, was identified as having the highest number of connections in the fecal co-occurrence network (33 positives and 29 negative correlations). This particular ASV is co-occurring with ASVs identified as *Prevotella*, *Blautia* and *Parabacteroides,* commonly found in the fecal microbiome and essential for polysaccharide degradation. However, ASV1 abundance was negatively correlated with other members of the Ruminococcaceae family, such as UCG-004, UCG-010 and UCG-005, which indicates a competition among species of this family. The others highly connected ASVs correspond to the genera *Parabacteroides* (ASV 36), *Blautia* (ASV 28) and an unknown genus from Lachnospiraceae family (ASV 62).

The archaea *M. gottschalkii* and *M. ruminantium* have different co-occurrence patterns with fibrolytic bacteria. *M. gottschalkii* co-occurred with bacteria from the genera *Prevotella*, *Succiniclasticum* and *Ruminococus*, while *M. ruminantium* co-occurred with bacteria from the family Lachnospiraceae and the genera *Papilibacter* and *Acetitomaculum*. Likewise, this difference was also observed in the fecal microbiome, in which the archaea *M. gottschalkii* co-occurred with bacteria from the genus *Bacteroides*, family Lachnospiraceae and Christensenellaceae, while *M. ruminantium* co-occurred with genera Ruminococcaceae UCG-005, *Marvinbryantia* and *Blautia*. Indeed, Kittelman et al. [13] demonstrated that the *M. gottschalkii* and *M. ruminantium* have different patterns of co-occurrence with fibrolytic bacteria in *Bos taurus*. However, this is the first time that this pattern is observed in the fecal microbiome.

The genera *Bozasella*/*Triplumaria* and *Entodinium*, the two dominant protozoan genera identified in Nelore, presented a strong and significant association with the archaea species *M. gottschalkii* and *M. ruminantium* and with fibrolytic bacteria, such as *Prevotella* and *Fibrobacter* [2]. Protozoa are known to be colonized by methanogens either as intracytoplasmic commensals or attached to their exterior surface [42, 46] and, although the symbiotic relationship between methanogens and protozoa is still unclear, our findings indicated a close relationship between these genera. Furthermore, the abundance of the genus *Fibrobacter* and some species of *Prevotella* are reduced in defaunated animals, suggesting that these genera may share a beneficial relationship with ciliate protozoa [47].

### Co-occurrence of ASVs inter-microbiomes indicates that fecal archaea as dependent on the rumen archaea population

Among the 96 bacterial ASVs identified as common to both microbiomes, only eight significant pairwise co-occurrences were found, but none between the same ASV. Therefore, this indicates that despite having a considerable number of ASVs in common, the fecal and ruminal populations of bacteria are not co-occurring. Indeed, Tapio et al. [10] reported similar results with a different approach, in which only the bacterial taxa present in oral samples are similar to rumen samples. Studies have shown that bacteria colonize and persist in different environments, including the transfer of gut microbiome from mice to zebrafish [48, 49]. This is evidence that the set of conditions in which these organisms can live, also known as the fundamental niche, is much larger than the conditions where the organism does live, or its realized niche [50]. The absence of co-occurrence of bacterial ASVs between microbiomes indicates that they successfully colonized both microbiomes, thus acquiring independent growth patterns according to each environmental condition.

On the other hand, of the 39/47 archaeal ASVs common to both environments co-occurred between microbiomes. Moreover, nine ruminal ASVs identified as *M. gottschalkii*, two as *M. ruminantium* and 4 as *Methanosphaera* had a strong and significant positive correlation with the same ASVs in the fecal microbiome. These co-occurrent ASVs represent 26.34% and 42.73% of the archaeal diversity in the rumen and feces microbiomes, respectively, which indicates that they are not typical residents of the gut/fecal environment but carried through the GIT. Also, these specific ruminal archaeal ASVs co-occurred with 19 highly abundant archaeal ASVs in the rumen, which corresponds to a total of 15.28% of the mean archaea abundance. Although new experiments should be performed to investigate further the relations between these microbiomes, such as shotgun metagenomic sequencing and quantitative PCR targeting these ASVs, these results indicate the potential use of the fecal archaea microbiome as a proxy for the ruminal archaea population in Nelore cattle.

## Conclusions

The rumen and fecal microbiomes harbor structured populations with abundant microorganisms, whose co-occurrences may reflect their relationships. Archaeal ASVs identified as co-occurring between microbiomes corresponded to a significant part of the overall archaeal diversity, which in fact, is much higher if one includes the interactions of these co-occurring ASVs with other archaea within their own microbiomes. Therefore, we suggest that fecal methanogens could be used as a proxy for rumen methanogens.

## Supplementary information

**Additional file 1:** **Figure S1.** A) Rarefaction curves of Bacterial 16S samples using the Shannon-Wiener index. B) Observed ASVs are significantly different among the environments when compared using the Kruskal-Wallis test. C) Shannon-Wiener index is significantly different among the environments when compared using the Kruskal-Wallis test. D) PCoA plot showing the stratification of the microbial populations in both environments.

**Additional file 2:** **Figure S2.** A) Rarefaction curves of Archaeal 16S samples using the Shannon-Wiener index. B) Observed ASVs are not significantly different among the environments when compared using the Kruskal-Wallis test. C) Shannon-Wiener index is not significantly different among the environments when compared using the Kruskal-Wallis test. D) PCoA plot showing the stratification of the microbial populations in both environments.

**Additional file 3:** **Figure S3.** A) Co-occurrence networks of bacterial ASVs identified in the ruminal microbiome. B) Co-occurrence networks of bacterial ASVs of the fecal. C) Co-occurrence networks of archaea ASVs identified in the ruminal microbiome. D) Co-occurrence networks of archaea ASVs identified in the fecal microbiome. ASVs are represented by their respective numbers and their taxonomic information, from family to order, by colors. Red edges represent positive correlation and blue, negative. Edges widths are related to the strength of the correlation.

**Additional file 4:** **Figure S4.** Co-occurrence networks of interdomain ASVs (Bacteria, Archaea and Protozoa). A) rumen and B) fecal origin, based on SparCC results. ASVs are represented by their respective numbers and their taxonomic information, from family to order, by colors. Red edges represent positive correlation and blue, negative. Edges widths are related to the strength of the correlation.

## Data Availability

All sequencing data are available in the NCBI Sequence Read Archive (SRA), under the bioproject number PRJNA525838.

## Abbreviations

ASV: Amplicon Sequence Variant
GIT: Gastrointestinal Tract
OTU: Operational Taxonomic Unit
