# Supplementary figures and images for: The structure of microbial populations in Nelore GIT reveals inter-dependency of methanogens in feces and rumen
Source: J Anim Sci Biotechnol. 2020 Feb 24;11:6. doi: 10.1186/s40104-019-0422-x (PMC7038601; doi:10.1186/s40104-019-0422-x)

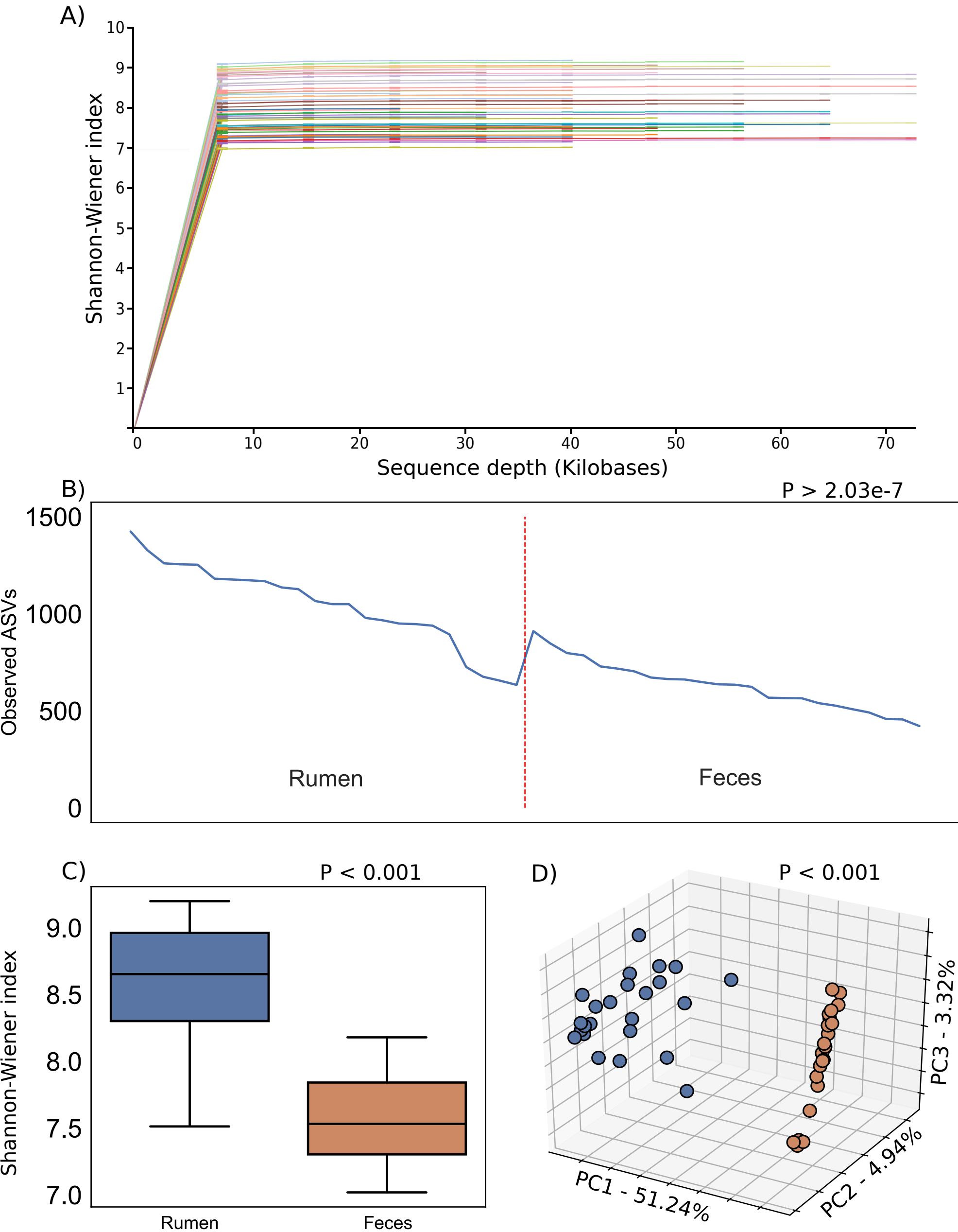

Supplement: Supplementary file 1 — Additional file 1: Figure S1. A) Rarefaction curves of Bacterial 16S samples using the Shannon-Wiener index. B) Observed ASVs are significantly different among the environments when compared using the Kruskal-Wallis test. C) Shannon-Wiener index is significantly different among the environments when compared using the Kruskal-Wallis test. D) PCoA plot showing the stratification of the microbial populations in both environments. [file 40104_2019_422_MOESM1_ESM.pdf]

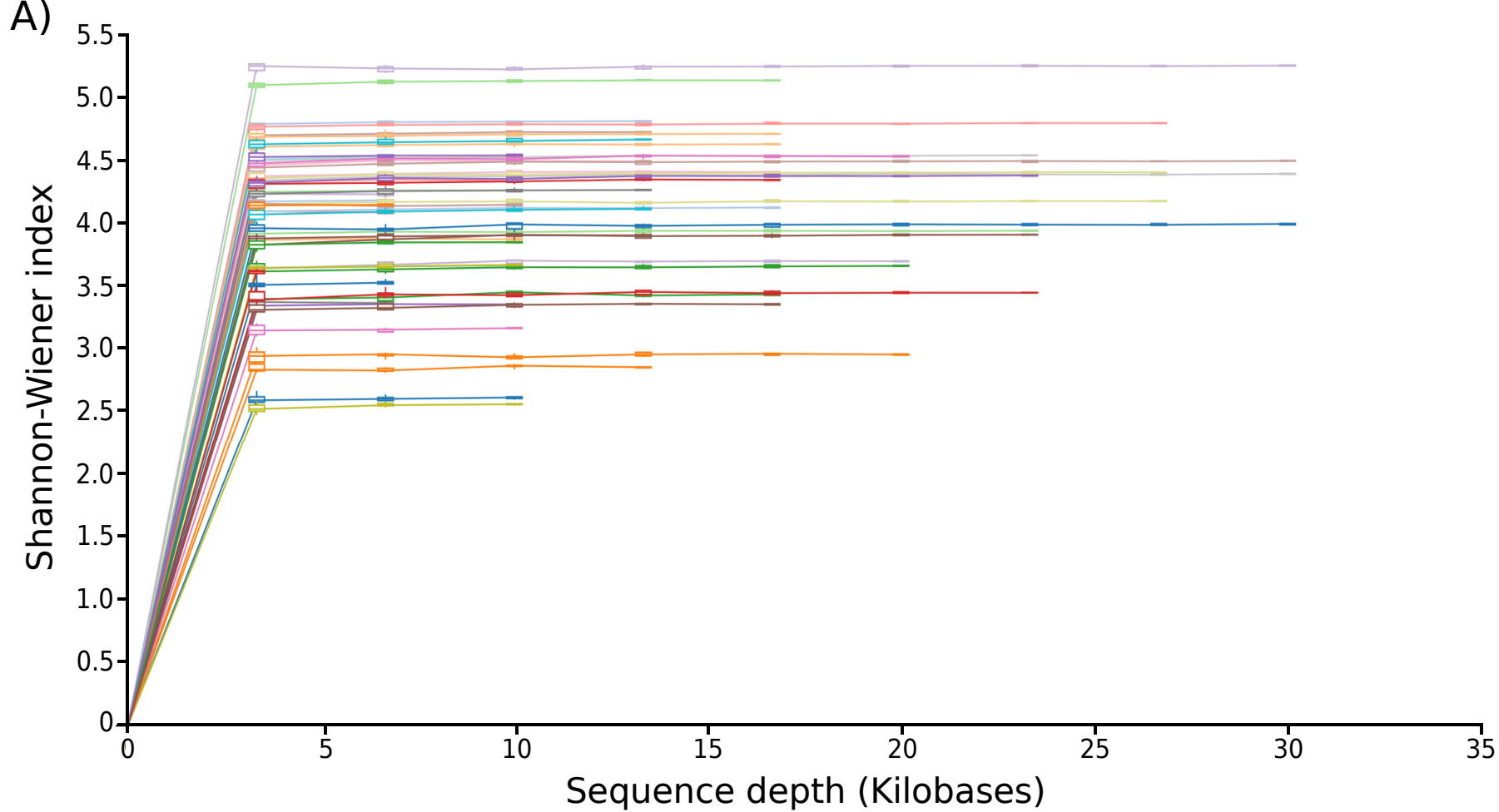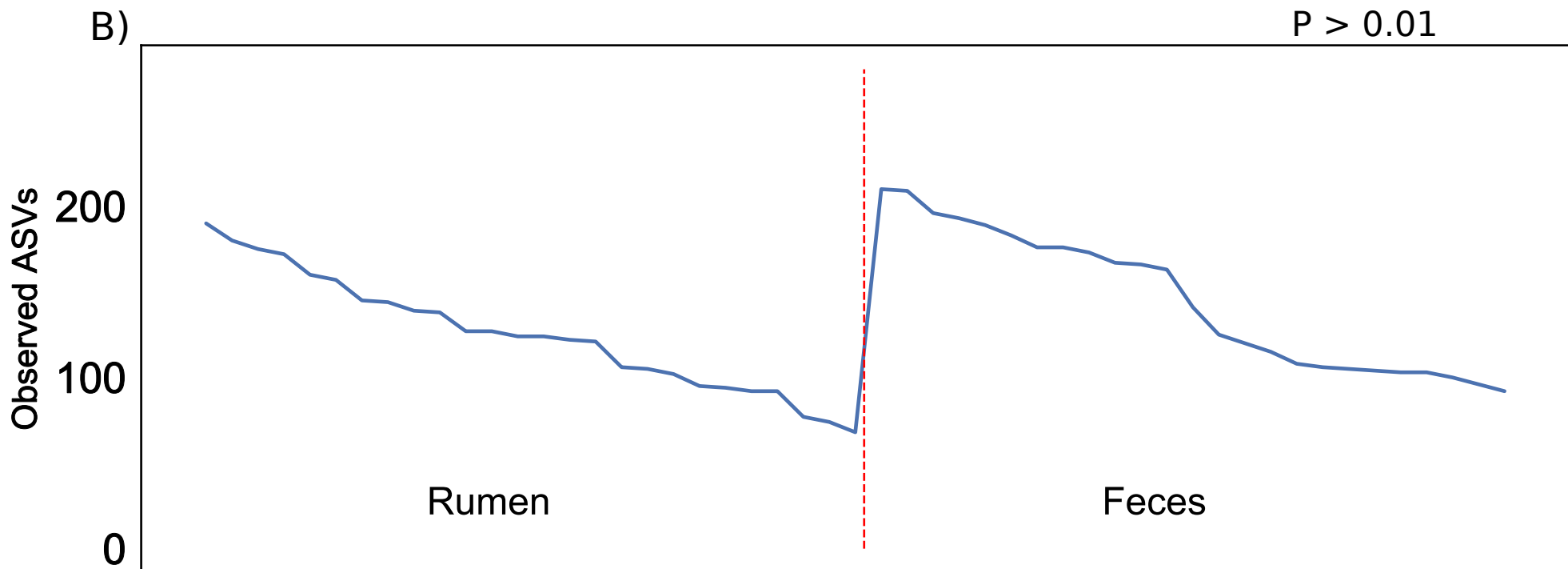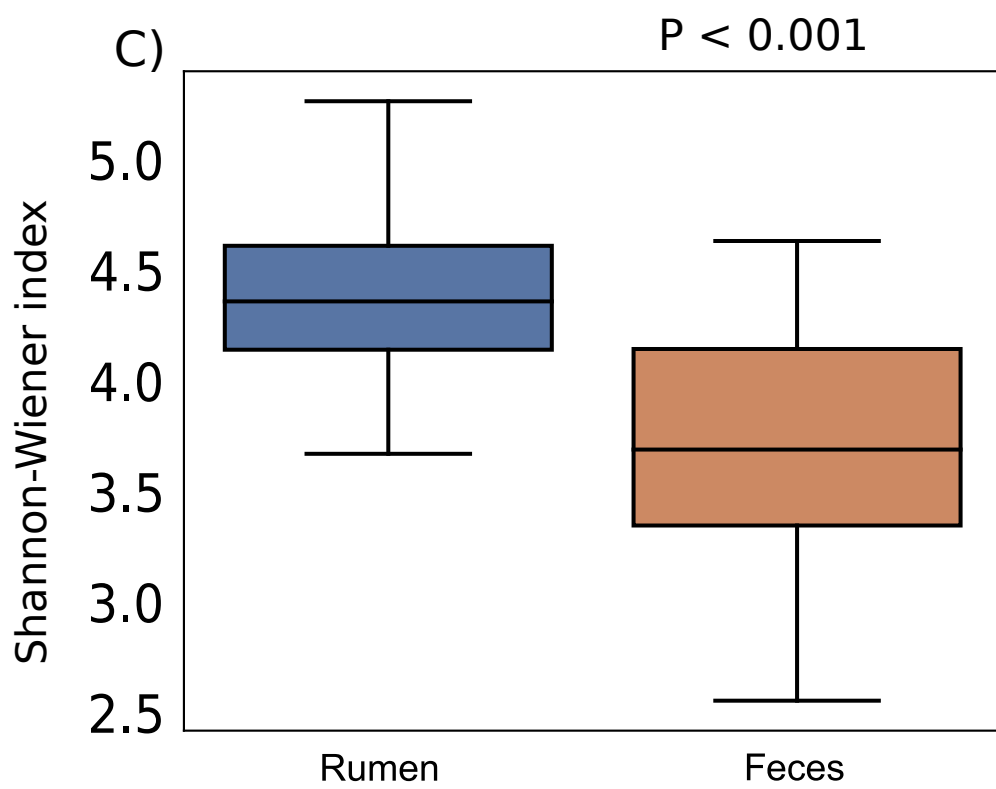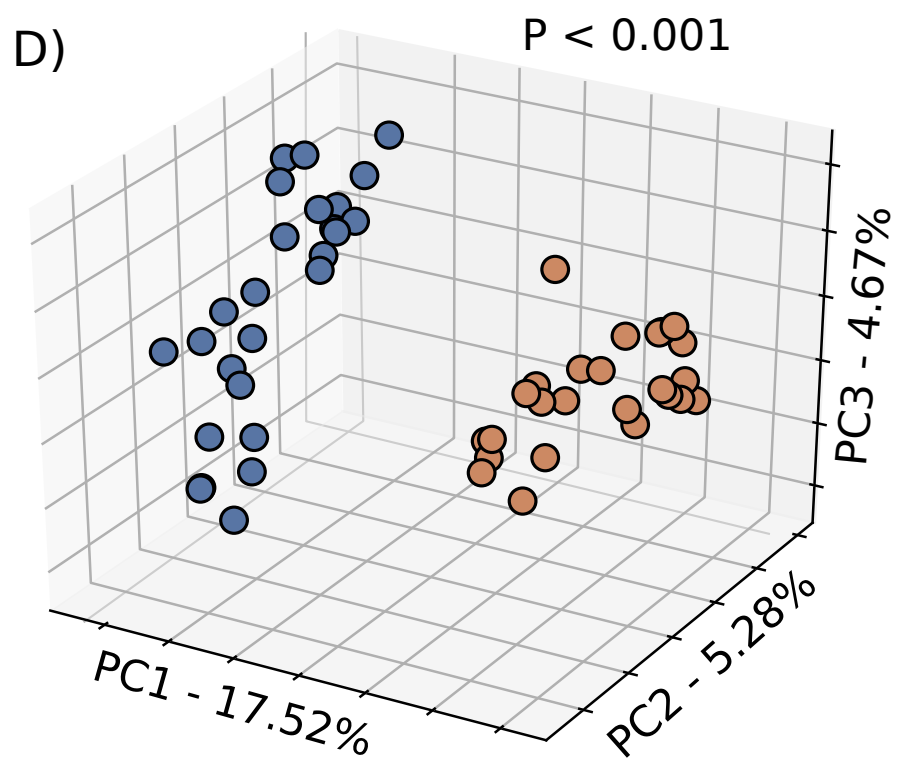

Supplement: Supplementary file 2 — Additional file 2: Figure S2. A) Rarefaction curves of Archaeal 16S samples using the Shannon-Wiener index. B) Observed ASVs are not significantly different among the environments when compared using the Kruskal-Wallis test. C) Shannon-Wiener index is not significantly different among the environments when compared using the Kruskal-Wallis test. D) PCoA plot showing the stratification of the microbial populations in both environments. [file 40104_2019_422_MOESM2_ESM.pdf]
