# Supplementary material for: The structure of microbial populations in Nelore GIT reveals inter-dependency of methanogens in feces and rumen
Source: J Anim Sci Biotechnol. 2020 Feb 24;11:6. doi: 10.1186/s40104-019-0422-x (PMC7038601; doi:10.1186/s40104-019-0422-x)
Supplement: Supplementary file 3 — Additional file 3: Figure S3. A) Co-occurrence networks of bacterial ASVs identified in the ruminal microbiome. B) Co-occurrence networks of bacterial ASVs of the fecal. C) Co-occurrence networks of archaea ASVs identified in the ruminal microbiome. D) Co-occurrence networks of archaea ASVs identified in the fecal microbiome. ASVs are represented by their respective numbers and their taxonomic information, from family to order, by colors. Red edges represent positive correlation and blue, negative. Edges widths are related to the strength of the correlation. [file 40104_2019_422_MOESM3_ESM.pdf]

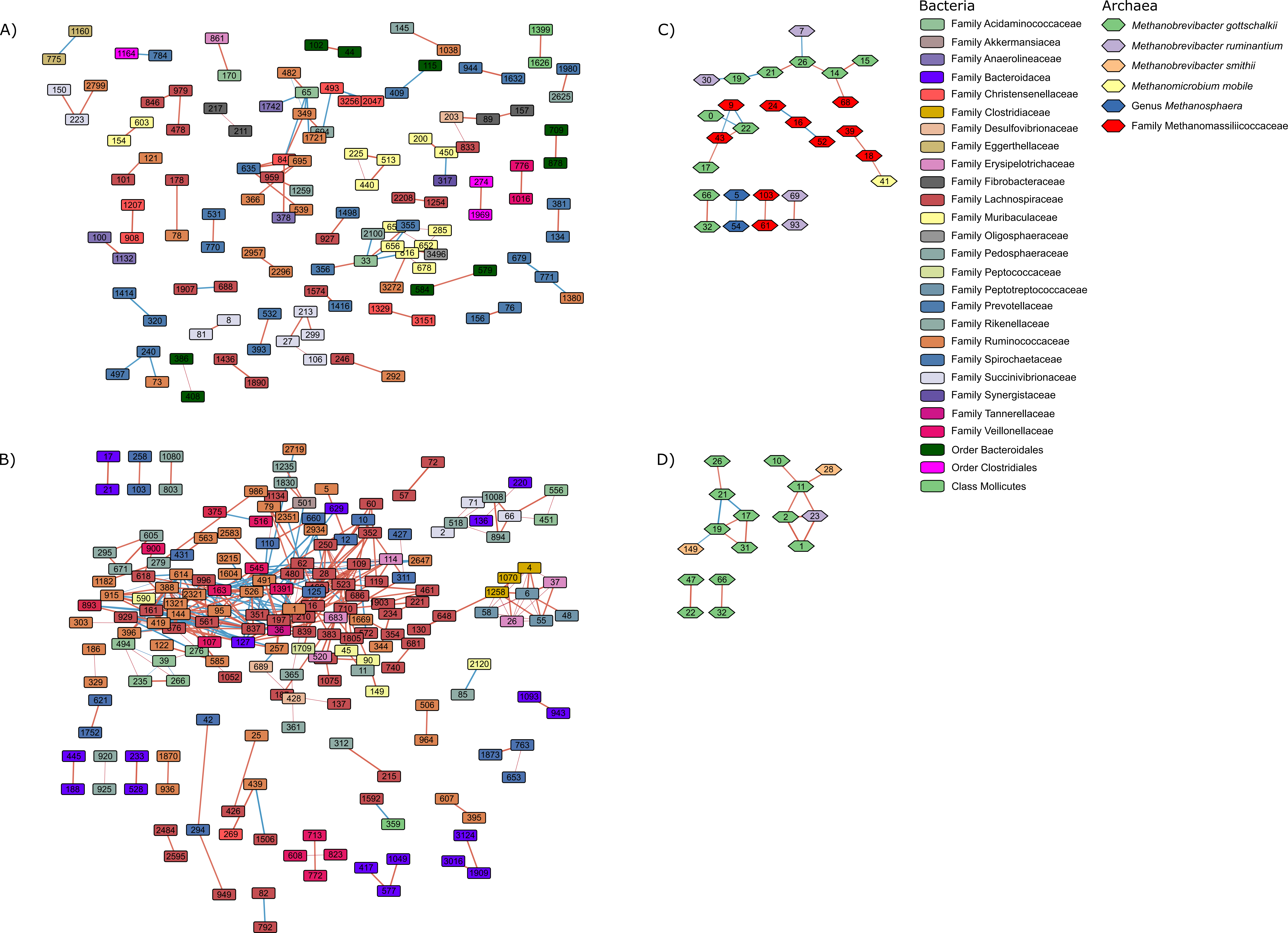

A)
B)
C)
D)
Bacteria
Family Acidaminococcaceae
Family Akkermansiacea
Family Anaerolineaceae
Family Bacteroidacea
Family Christensenellaceae
Family Clostridiaceae
Family Desulfovibrionaceae
Family Eggerthellaceae
Family Erysipelotrichaceae
Family Fibrobacteraceae
Family Lachnospiraceae
Family Muribaculaceae
Family Oligosphaeraceae
Family Pedosphaeraceae
Family Peptococcaceae
Family Peptotreptococcaceae
Family Prevotellaceae
Family Rikenellaceae
Family Ruminococcaceae
Family Spirochaetaceae
Family Succinivibrionaceae
Family Synergistaceae
Family Tannerellaceae
Family Veillonellaceae
Order Bacteroidales
Order Clostridiales
Class Mollicutes
Archaea
Methanobrevibacter gottschalkii
Methanobrevibacter ruminantium
Methanobrevibacter smithii
Methanomicrobium mobile
Genus Methanosphaera
Family Methanomassiliicoccaceae
