# Supplementary material for: The structure of microbial populations in Nelore GIT reveals inter-dependency of methanogens in feces and rumen
Source: J Anim Sci Biotechnol. 2020 Feb 24;11:6. doi: 10.1186/s40104-019-0422-x (PMC7038601; doi:10.1186/s40104-019-0422-x)
Supplement: Supplementary file 4 — Additional file 4: Figure S4. Co-occurrence networks of interdomain ASVs (Bacteria, Archaea and Protozoa). A) rumen and B) fecal origin, based on SparCC results. ASVs are represented by their respective numbers and their taxonomic information, from family to order, by colors. Red edges represent positive correlation and blue, negative. Edges widths are related to the strength of the correlation. [file 40104_2019_422_MOESM4_ESM.pdf]

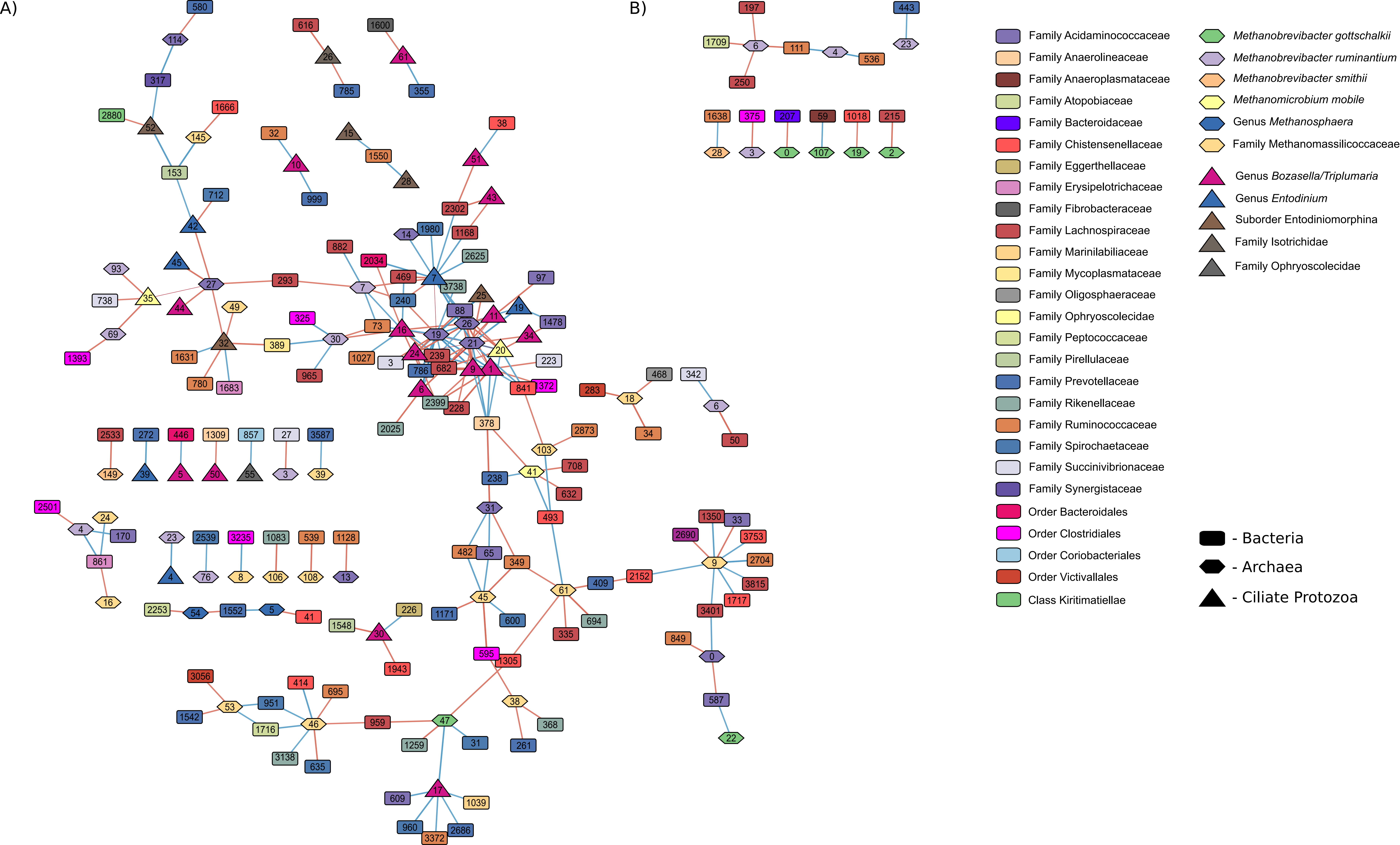
A)
B)
Family Acidaminococcaceae
Family Anaerolineaceae
Family Anaeroplasmataceae
Family Atopobiaceae
Family Bacteroidaceae
Family Chistensenellaceae
Family Eggerthellaceae
Family Erysipelotrichaceae
Family Fibrobacteraceae
Family Lachnospiraceae
Family Marinilabiliaceae
Family Mycoplasmataceae
Family Oligosphaeraceae
Family Ophryoscolecidae
Family Peptococcaceae
Family Pirellulaceae
Family Prevotellaceae
Family Rikenellaceae
Family Ruminococcaceae
Family Spirochaetaceae
Family Succinivibrionaceae
Family Synergistaceae
Order Bacteroidales
Order Clostridiales
Order Coriobacteriales
Order Victivallales
Class Kiritimatiellae
Methanobrevibacter gottschalkii
Methanobrevibacter ruminantium
Methanobrevibacter smithii
Methanomicrobium mobile
Genus *Methanosphaera*
Family Methanomassilicoccaceae
Genus *Bozasella/Triplumaria*
Genus *Entodinium*
Suborder Entodiniomorphina
Family Isotrichidae
Family Ophryoscolecidae
- Bacteria
- Archaea
- Ciliate Protozoa
